# The impact of dehydroepiandrosterone in poor ovarian responders on assisted reproduction technology treatment

**DOI:** 10.5935/1518-0557.20190045

**Published:** 2019

**Authors:** Cecilia de Souza Monteiro, Bruno Brum Scheffer, Rafaela Friche de Carvalho, Juliano Brum Scheffer

**Affiliations:** 1 Brazilian Institute of Assisted Reproduction (IBRRA), Belo Horizonte, MG, Brazil

**Keywords:** dehydroepiandrosterone, poor ovarian responder, Bologna criteria, antral follicle count, anti-Müllerian hormone, *in vitro* fertilization

## Abstract

One in six couples worldwide will experience at least one infertility problem during their reproductive years. Between 5.6% and 35.1% of women will exhibit poor ovarian response. A variety of methods have been applied to improve ovarian response, including dehydroepiandrosterone. In the ovaries, dehydroepiandrosterone promotes follicular development and granulosa cell proliferation by increasing intraovarian androgen concentrations while simultaneously enhancing the level of follicular insulin-like growth factor-1, which promotes folliculogenesis. Dehydroepiandrosterone supplementation may improve *in vitro* fertilization outcomes and ovarian response in patients with poor ovarian response. However, a few questions still loom over the effectiveness of dehydroepiandrosterone.

## INTRODUCTION

One in six couples worldwide will experience at least one infertility problem during their reproductive years (ESHRE, 2018) and the majority may benefit from assisted reproduction technology (ART) treatments. Between 5.6% and 35.1% of women will exhibit poor ovarian response (POR) (Oudendijk *et al.*, 2012). Zhang *et al.* (2016) estimated that 5-18% of all *in vitro* fertilization (IVF) cycles are canceled for poor ovarian response.

The European Society of Human Reproduction and Embryology (ESHRE) standardized the criteria for poor ovarian response in the Bologna criteria. At least two out of three following criteria are required to define poor ovarian response during IVF: 1) Maternal age > 40 or any other risk factor for poor ovarian response; 2) prior poor ovarian response (≤ 3 oocytes with a conventional stimulation protocol); 3) abnormal ovarian reserve test (antral follicle count [AFC] less than 5-7 follicles or anti-Müllerian hormone [AMH] less than 0.5- 1.1ng/ml). The ESHRE also indicated that history of two episodes of poor ovarian response after a maximum stimulation protocol is enough to define poor ovarian response (Qin *et al.,* 2017).

A variety of methods have been applied to improve ovarian response, including increased gonadotropin dosage, modulation with gonadotropin-releasing hormone (GnRH), flare-up regimes, adjunctive human growth hormone therapy, minimal ovarian stimulation with clomiphene citrate, and unstimulated or natural cycle IVF. However, the outcomes of these treatments have been less than satisfactory (Wiser *et al.,* 2010).

Dehydroepiandrosterone (DHEA) is an endogenous steroid produced in the zona reticularis of the adrenal cortex and by ovarian theca cells. In the ovary, it promotes follicular development and granulosa cell proliferation by increasing intraovarian androgen concentrations. DHEA also enhances the level of follicular insulin-like growth factor-1 (IGF-1), which promotes folliculogenesis by enhancing the effect of gonadotropin and reducing follicular regression (Wiser *et al.,* 2010).

Despite the wider use of DHEA in poor responders, views among clinicians vary considerably. The aim of this study was to review the potential beneﬁts of DHEA supplementation for poor responders undergoing ART treatment.

## MATERIALS AND METHODS

Searches were carried out on PubMed and the Cochrane Library for relevant literature on the efficacy of DHEA at improving the ovarian response of women with poor ovarian response and women with premature ovarian aging after failed IVF. In all cases, DHEA supplementation was administered before ovarian stimulation in IVF cycles.

The following keywords were used in the searches: "DHEA"; "dehydroepiandrosterone"; "poor ovarian responder"; "low response"; "diminished ovarian reserve"; "IVF"; and "ICSI".

Randomized controlled trials (RCT), meta-analyses, systematic reviews, retrospective and prospective controlled studies were eligible for inclusion.

Our manual and automatic searches yielded a total of 38 publications. The full texts of the articles were retrieved, and 25 met the inclusion criteria. The selected articles were designed to compare whether pre-treatment with DHEA improved the IVF outcomes of patients with poor ovarian response.

In order to facilitate the understanding of the effects of DHEA supplementation on the response to IVF, we separated the results into topics as follows: effect of DHEA on ovarian reserve markers; effect of DHEA on response to ART treatment; effect of DHEA on oocyte quality; effect of DHEA on pregnancy/live birth rates; and other therapies for poor responders.

## RESULTS

### DHEA and ovarian reserve markers

The meta-analysis published by Zhang *et al.* (2016) investigated the AMH levels and the AFC of the same patients before and after DHEA supplementation. Only one of the analyzed studies was an RCT. The authors of the included articles did not report the administered doses of DHEA. The study population featured women with diminished ovarian reserve undergoing ovarian stimulation in IVF protocols. Six self-control studies indicated that DHEA treatment increased the AFC, with no significant heterogeneity observed (95% CI 0.14-0.66, *p*=0.002, I^2^=24%). Two articles described significant increases in AMH levels for all age groups after DHEA supplementation (95% CI 1.27-1.6, *p*<0.0001, I^2^=0%).

Conversely, the randomized double blind placebo-controlled pilot study by Yeung *et al.* (2014) did not detect statistically significant differences in the median AFC of individuals given DHEA compared with placebo (3.5 [1.75-4.25] *vs.* 4 [3-4]; *p*=0.436). This study included individuals with ages ≤ 40 years and subfertility lasting for more than a year, with poor ovarian response defined as an AFC < 5. Patients with a history of ovarian cystectomy or oophorectomy, individuals previously submitted to cytotoxic chemotherapy or pelvic irradiation, and subjects with a history of taking testosterone supplementation were excluded. The patients were given 25mg of DHEA three times a day, starting at least 12 weeks before the scheduled IVF treatment.

Similarly to the AFC, serum follicle stimulating hormone (FSH), AMH, and serum testosterone (cutoff at 1.0ng/ml) levels were not statistically different between the groups throughout the study period (Yeung *et al.*, 2014). This same study found that women with higher follicular DHEA levels (cutoff at 180µg/dL) had a statistically higher number of good-quality embryos (1 [0-2] *vs.* 0 [0-0.25]; *p*=0.013). Therefore, DHEA supplementation may have improved the ovarian environment in which follicular maturation takes place, leading to decreased aneuploidy. However, the underlying mechanism is still unknown.

Wiser *et al.* (2010) carried out a randomized prospective controlled study to evaluate the effects of DHEA supplementation on IVF. The case group received 75 mg of DHEA once a day at least six weeks prior to the IVF cycle and during treatment. Patients with prior poor response to ovarian stimulation were included in the study. Patients over the age of 42 and patients given DHEA at any time before the start of the study were excluded. The mean peak estradiol levels of the DHEA and control groups on the day of chorionic gonadotropin hormone (HCG) administration were not statistically different (732pg/ml and 917pg/ml, respectively; *p*=0.2).

Therefore, according to the literature, the effect of DHEA supplementation on ovarian reserve markers is still controversial. More trials are needed to understand the potential effects of DHEA on ovarian reserve markers and pregnancy hormones.

### DHEA and response to ART treatment

A meta-analysis by Li *et al.* (2015) concluded that DHEA did not increase the number of oocytes retrieved among poor responders submitted to IVF (95% CI 1.43 to 0.96; *p*=0.70). Participants in the case group received 75mg of DHEA daily for at least 12 weeks.

Zhang *et al.* (2016) published a meta-analysis and found three trials including 69 patients in which women aged less than 36 years had significant increases in the number of oocytes after treatment with DHEA (95% CI 2.15-2.61, *p*<0.0001, I^2^=1%). In a study included in this meta-analysis, Jirge *et al*. (2014) reported that the mean of number of retrieved oocytes from controls was 2.09, *versus* 4.45 in the DHEA group (RR 2.36 [95% CI 2.13-2.59]).

In this meta-analysis, six trials including 163 female patients aged 36+ years also indicated increased numbers of oocytes after DHEA treatment (95% CI 0.73-1.90, *p*<0.0001, I^2^=72%). The study by Tsui *et al*. (2015), for example, reported that the mean of number of retrieved oocytes from controls was 2.4, *versus* 4.2 in the DHEA group (RR 1.80 [95% CI 1.22-2.38]).

Although statistically heterogeneous, three case-control trials, two RCTs, and one prospective cohort study featured in this meta-analysis enrolling a combined 1398 patients were performed to determine implantation rates. The implantation rate of patients treated with DHEA was significantly higher than the rates seen in untreated controls (RR 1.56, 95% CI 1.20-2.01, *p*=0.0007). The study by Xu *et al.* (2014) reported that the implantation rate increased in DHEA group, from 27 to 47 patients (RR 1.82 [95% CI 1.19 -2.87]) (Zhang *et al.,* 2016).

Qin *et al.* (2017) published a meta-analysis to evaluate the effect of DHEA therapy on the ovarian response and pregnancy outcomes of patients with diminished ovarian reserve. Nine studies were included - four *RCTs, four retrospective studies, and one prospective study. The patients were given 25 mg of DHEA three times a week for a minimum of six or 12 weeks.* In this meta-analysis, two RCTs and three retrospective studies concluded that the number of retrieved oocytes was not different between the DHEA and control groups. There was significant heterogeneity between the studies (RR -0.69, 95% CI:2.18-0.81)

The patients included in a randomized controlled trial by Kotb *et al.* (2016) were given 25 mg of DHEA three times daily for three months before IVF. Women undergoing IVF with POR based on the Bologna criteria with ages ranging from 20 to 45 years were included. Women with a body mass index > 35 kg/m^2^, individuals with a single ovary, subjects allergic to DHEA, and females with diabetes were excluded. The individuals given DHEA had statistically greater counts of retrieved oocytes (6.9 *vs.* 3.1, *p*=0.039), significantly higher fertilization rates (62.3% *vs.* 52.2%, *p*=0.03), fewer days on controlled ovarian hyperstimulation (11.6 *vs.* 12.6, *p*=0.001), and lower gonadotropin doses (3383IU *vs.* 3653IU, *p*=0.045). Interestingly, the number of metaphase II (MII) oocytes, total number of embryos, and number of transferred embryos were not different in the case groups (Kotb *et al.,* 2016).

The literature indicates the existence of a tendency in DHEA supplementation increasing the number of oocytes retrieved and embryo implantation rates. More randomized controlled trials are needed to confirm the actual benefits of DHEA for patients with poor ovarian response undergoing ART treatment.

### DHEA and oocyte quality

Yeung *et al.* (2014) concluded that the number of follicles and oocytes retrieved were similar between groups, although the DHEA group had a non-statistically greater mean number of fertilized embryos (1 *vs.* 3, *p*=0.155), cleaved embryos (1 *vs.* 3, *p*=0.169), transferred embryos (1 *vs.* 2, *p*=0.430), and top-quality embryos (0 *vs.* 1, *p*=0.141).

The retrospective analysis by Ferrario *et al*. (2015) found that AMH and DHEA sulfate levels were positively correlated with the number of mature oocytes (RR=0.784), fertilized oocytes (RR=0.607), and developed embryos (RR=0,513). The authors retrospectively analyzed the data from 148 poor responders diagnosed based on the Bologna criteria. Women with polycystic ovary syndrome and endometriosis were excluded, along with normal responders and patients with severe male factor (cryptozoospermia or azoospermia).

Therefore, few studies have investigated the correlation between DHEA and oocyte quality, and the findings are still controversial.

### DHEA and pregnancy/live birth rates

In the meta-analysis by Li *et al.* (2015), six studies evaluated the effects of DHEA on clinical pregnancy rates. DHEA significantly increased the clinical pregnancy rates of poor responders submitted to IVF compared with controls (RR 2.13, 95% CI 1.12-4.08; *p*=0.02). However, no significant effect was observed in an analysis including only RCTs. In addition, careful evaluation of each individual study included in the meta-analysis found that most of the studies were not statistically sound.

The meta-analysis of Zhang *et al.* (2016) included eight randomized control trials, ten cohort studies, and three case-control studies designed to calculate clinical pregnancy rates. The meta-analysis found statistically significant increases in the pregnancy rates of patients treated with DHEA (RR 1.53, 95% CI 1.25-1.86, *p*<0.0001). However, most of the studies were not statistically sound.

The meta-analysis cited above included four RCTs (Moawad & Shaeer*,* 2012; Tartagni *et al.,* 2015; Wiser *et al.,* 2010; Yeung *et al.,* 2014) and two prospective cohort trials (Jirge *et al.,* 2014; Vlahos *et al.,* 2015) designed to calculate live birth rates. In the group analysis of these articles, there were significantly higher live birth rates among the patients given DHEA (RR 1.87, 95% CI 1.22-2.88, *p*=0.004) (Zhang *et al.,* 2016). However, careful evaluation of each individual study found that only one (Tartagni *et al.,* 2015) reported statistically reliable higher live birth rates in the DHEA group (RR 1.79, 95% CI 1.01-3.17). In this randomized study, 109 infertile patients with ages ranging from 36-40 years were selected to undergo the long protocol and received 75 mg of DHEA once a day for eight weeks before starting IVF.

The meta-analysis by Qin *et al.* (2017) included four RCTs (Wiser *et al.,* 2010; Kara *et al.,* 2014; Zhang *et al.,* 2014; Yeung *et al.,* 2014), three retrospective studies (Barad *et al.,* 2007; Xu *et al.,* 2014; Fusi *et al*., 2013), and one prospective study (Vlahos *et al.,* 2015) designed to analyze clinical pregnancy rates. Clinical pregnancy rates were significantly higher in the DHEA group (OR 1.47, 95% CI: 1.09-1.99). However, subgroup analysis based on RCTs revealed that there was no significant difference between the groups (OR 1.08, 95% CI: 0.67-1.73).

The randomized controlled trial by Kotb *et al*. (2016) described significant benefits from DHEA to poor responders in the form of increased clinical pregnancy (11 *vs.* 23, *p*=0.09) and ongoing pregnancy (9 *vs.* 20, *p*=0.036) rates.

The randomized controlled trial by Yeung *et al*. (2014) failed to identify statistically significant differences in clinical pregnancy (18.8% *vs.* 25.0%, *p*=0.380), ongoing pregnancy (18.8% *vs.* 12.5%, *p*=0.326), live birth (12.5% *vs.* 12.5%, *p*=1.0), or miscarriage (0 *vs.* 12.5%, *p*=0.326) rates.

The randomized prospective study by Wiser *et al*. (2010) described higher clinical pregnancy (2 *vs.* 4, *p*=0.25) and live birth (1 *vs.* 3, *p*=0.20) rates among individuals given DHEA. However, the differences were not statistically significant.

The literature describes a tendency toward increased clinical pregnancy and live birth rates in females given DHEA, albeit not statistically significant.

### Other therapies for poor responders

In addition to DHEA, the agents more commonly used in daily practice to improve ovarian response in IVF cycles are transdermal testosterone, clomiphene citrate, aromatase inhibitors, recombinant LH, and recombinant human chorionic gonadotropin.

The meta-analysis by González-Comadran *et al.* (2012) looked into the effects of transdermal testosterone in women with poor ovarian response undergoing IVF. The authors focused on transdermal testosterone, an agent known to produce powerful systemic androgenization and subsequent greater action of FSH compared with other androgen-modulating agents. Three randomized controlled trials were included. The dose of transdermal testosterone used in the intervention group varied between the articles as follows: gel, 10 mg for 15-20 days; gel, 12.5 mg for 21 days during pituitary desensitization; or 2.5 mg patches per day for five days.

The authors also found that women on transdermal testosterone achieved significantly higher live birth rates (RR 1.91, 95%CI 1.01-3.63, I^2^=0%) (González-Comadran *et al.,* 2012).

The systematic review by Kamath *et al.* (2017) analyzed the effectiveness of oral medication for ovulation induction (clomiphene citrate, 100mg daily for five days; letrozole, 5 mg daily for five days) versus gonadotropin-only regimens for controlled ovarian stimulation in IVF. The authors were unable to find conclusive evidence indicating that clomiphene citrate or letrozole with gonadotropins differed from gonadotropin-only in terms of live birth or pregnancy rates, either in the general population of women undergoing IVF cycles or in poor responders.

Large, high quality controlled randomized trials are needed to provide input on others therapies designed to improve the IVF outcomes of poor responders.

## DISCUSSION

This review aimed to evaluate whether DHEA supplementation might increase the ovarian response of females undergoing IVF cycles. The first obstacle to attaining the goal of this review was to find consensus over the definition of POR. Authors define POR based on different principles and use of the Bologna criteria is not universal. Secondly, the populations selected in each study were not similar in matters such as age, cause of infertility, stimulation protocols, or total dose of ovulation induction medication. And lastly, there was no standardization over the time of administration or dose of DHEA in use in the studies.

Despite substantial heterogeneity between studies, we have decided to review the current literature on DHEA and ovarian response. And based on this review, DHEA supplementation appears to improve the IVF outcomes and level of ovarian response of poor responders. DHEA is a simple-to-use, side-effect-free medication probably linked to fewer days of stimulation and lower gonadotropin doses. Furthermore, according to our review, DHEA showed a tendency toward increased numbers of retrieved oocytes and higher embryo implantation, clinical pregnancy, and live birth rates. Further randomized controlled trials are needed to confirm the actual benefits of DHEA to patients with poor ovarian response.

## CONCLUSIONS

Few RCTs have looked into DHEA supplementation as adjuvant therapy in IVF cycles. Further multicenter RCTs are needed to confirm whether supplementation with DHEA improves the outcomes of poor responders.
